# Supplementary material for: Empirical methods for controlling false positives and estimating confidence in ChIP-Seq peaks
Source: BMC Bioinformatics. 2008 Dec 5;9:523. doi: 10.1186/1471-2105-9-523 (PMC2628906; doi:10.1186/1471-2105-9-523)
Supplement: Additional File 1 — A variety of html documents from the USeq web site detailing the available applications, their best usage, output file type descriptions, command line menus, etc. [file 1471-2105-9-523-S1.zip › USeqUserGuides/JavaDoc.zip!/JavaDoc/cgar/Util.html]

Util


|  |  |  |
| --- | --- | --- |
| **Overview** \| **Package** \| **Class** \| **Use** \| **Tree** \| **Deprecated** \| **Index** \| **Help** | |  |
| **PREV CLASS**   NEXT CLASS | **FRAMES**    **NO FRAMES**     **All Classes** |  |
| SUMMARY: NESTED \| FIELD \| CONSTR \| METHOD | DETAIL: FIELD \| CONSTR \| METHOD |  |


---


## cgar Class Util

```
java.lang.Object
  cgar.Util
```

---

``` public class Util extends java.lang.Object ```

---

| **Field Summary** | |
| --- | --- |
| `static java.lang.String` | `baseURL` |
| `static java.lang.String` | `contactEmail` |
| `static java.lang.String` | `databaseDriver` |
| `static java.lang.String` | `databaseName` |
| `static java.lang.String` | `databasePassword` |
| `static java.lang.String` | `databaseURL` |
| `static java.lang.String` | `databaseUser` |


**Constructor Summary**

`Util()`


| **Method Summary** | |
| --- | --- |
| `boolean` | `checkIP(java.lang.String IP)`             Checks to see if a given IP has exceeded the number of allowable failed logins. |
| `java.lang.String[]` | `fetchLabGroupNames()`             Returns LabGroup.name, initiates an sql connection and the closes it so don't call if you are doing anything else. |
| `SQL` | `getSQL()`             Make connection to CGAR DB. |
| `void` | `updateLoginFailure(java.lang.String IP)`             Increments the numberFailures for an ip in the Login table if it exists or creates a new record. |

**Methods inherited from class java.lang.Object**

`equals, getClass, hashCode, notify, notifyAll, toString, wait, wait, wait`

**Field Detail**

### databaseName

```
public static final java.lang.String databaseName
```

**See Also:**: Constant Field Values

---


### databaseUser

```
public static final java.lang.String databaseUser
```

**See Also:**: Constant Field Values

---


### databasePassword

```
public static final java.lang.String databasePassword
```

**See Also:**: Constant Field Values

---


### databaseURL

```
public static final java.lang.String databaseURL
```

**See Also:**: Constant Field Values

---


### databaseDriver

```
public static final java.lang.String databaseDriver
```

**See Also:**: Constant Field Values

---


### contactEmail

```
public static final java.lang.String contactEmail
```

**See Also:**: Constant Field Values

---


### baseURL

```
public static final java.lang.String baseURL
```

**See Also:**: Constant Field Values


**Constructor Detail**

### Util

```
public Util()
```


**Method Detail**

### fetchLabGroupNames

```
public java.lang.String[] fetchLabGroupNames()
```

:   Returns LabGroup.name, initiates an sql connection and the closes it so don't call if
    you are doing anything else.

---


### getSQL

```
public SQL getSQL()
```

:   Make connection to CGAR DB.
    Dont' forget to close this connection when done with sql queries!

---


### checkIP

```
public boolean checkIP(java.lang.String IP)
```

:   Checks to see if a given IP has exceeded the number of allowable failed logins.
    Will create a new IP entry if it's not found. Will reset entry and return true
    if 12hrs has passed.

---


### updateLoginFailure

```
public void updateLoginFailure(java.lang.String IP)
```

:   Increments the numberFailures for an ip in the Login table if it exists or
    creates a new record.


---


|  |  |  |
| --- | --- | --- |
| **Overview** \| **Package** \| **Class** \| **Use** \| **Tree** \| **Deprecated** \| **Index** \| **Help** | |  |
| **PREV CLASS**   NEXT CLASS | **FRAMES**    **NO FRAMES**     **All Classes** |  |
| SUMMARY: NESTED \| FIELD \| CONSTR \| METHOD | DETAIL: FIELD \| CONSTR \| METHOD |  |


---
